# JAK inhibitors remove innate immune barriers facilitating viral propagation

**DOI:** 10.1093/narmme/ugaf017

**Published:** 2025-05-02

**Authors:** Erlend Ravlo, Aleksandr Ianevski, Marius Nårstad Skipperstøen, Hilde Lysvand, Jørn-Ove Schjølberg, Ole Solheim, Wei Wang, Miroslava Kissova, Marthe Vestvik, Olli Vapalahti, Teemu Smura, Hanna Vauhkonen, Valentyn Oksenych, Friedemann Weber, Mårten Strand, Magnus Evander, Janne Fossum Malmring, Jan Egil Afset, Magnar Bjørås, Denis E Kainov

**Affiliations:** Department of Clinical and Molecular Medicine (IKOM), Norwegian University of Science and Technology, 7028 Trondheim, Norway; Department of Clinical and Molecular Medicine (IKOM), Norwegian University of Science and Technology, 7028 Trondheim, Norway; Department of Clinical and Molecular Medicine (IKOM), Norwegian University of Science and Technology, 7028 Trondheim, Norway; Department of Clinical and Molecular Medicine (IKOM), Norwegian University of Science and Technology, 7028 Trondheim, Norway; Department of Clinical and Molecular Medicine (IKOM), Norwegian University of Science and Technology, 7028 Trondheim, Norway; Department of Microbiology, Oslo University Hospital and University of Oslo, 0372 Oslo, Norway; Department of Clinical and Molecular Medicine (IKOM), Norwegian University of Science and Technology, 7028 Trondheim, Norway; Department of Clinical and Molecular Medicine (IKOM), Norwegian University of Science and Technology, 7028 Trondheim, Norway; Department of Clinical and Molecular Medicine (IKOM), Norwegian University of Science and Technology, 7028 Trondheim, Norway; Department of Clinical and Molecular Medicine (IKOM), Norwegian University of Science and Technology, 7028 Trondheim, Norway; Department of Virology, University of Helsinki, 00014 Helsinki, Finland; HUS Diagnostic Center, Clinical Microbiology, Helsinki University Hospital, University of Helsinki, 00029 Helsinki, Finland; Department of Virology, University of Helsinki, 00014 Helsinki, Finland; HUS Diagnostic Center, Clinical Microbiology, Helsinki University Hospital, University of Helsinki, 00029 Helsinki, Finland; Department of Virology, University of Helsinki, 00014 Helsinki, Finland; HUS Diagnostic Center, Clinical Microbiology, Helsinki University Hospital, University of Helsinki, 00029 Helsinki, Finland; Department of Clinical and Molecular Medicine (IKOM), Norwegian University of Science and Technology, 7028 Trondheim, Norway; Institute for Virology, FB10-Veterinary Medicine, D-35392 Gießen, Germany; Department of Clinical Microbiology, Virology, Umeå University, 90185 Umeå, Sweden; Department of Clinical Microbiology, Virology, Umeå University, 90185 Umeå, Sweden; Department of Medical Microbiology, Clinic for Laboratory Medicine, St. Olavs Hospital, 7028 Trondheim, Norway; Department of Clinical and Molecular Medicine (IKOM), Norwegian University of Science and Technology, 7028 Trondheim, Norway; Department of Medical Microbiology, Clinic for Laboratory Medicine, St. Olavs Hospital, 7028 Trondheim, Norway; Department of Clinical and Molecular Medicine (IKOM), Norwegian University of Science and Technology, 7028 Trondheim, Norway; Department of Microbiology, Oslo University Hospital and University of Oslo, 0372 Oslo, Norway; Centre for Embryology and Healthy Development (CRESCO), University of Oslo, Oslo 0373, Norway; Department of Clinical and Molecular Medicine (IKOM), Norwegian University of Science and Technology, 7028 Trondheim, Norway; Institute for Molecular Medicine FIMM, Helsinki Institute for Life Science, University of Helsinki, 00014 Helsinki Finland

## Abstract

Janus kinase (JAK) inhibitors are small-molecule therapeutics that reduce inflammation in autoimmune and inflammatory diseases by modulating the JAK–STAT pathway. While effective in alleviating immune-mediated conditions, JAK inhibitors can impair antiviral defences by suppressing interferon (IFN) responses, potentially increasing susceptibility to viral infections. This study investigates the pro-viral mechanism of JAK inhibitors, focusing on baricitinib, across various cell lines, organoids, and viral strains, including a recombinant Rift Valley fever virus, influenza A virus, SARS-CoV-2, and wild-type adenovirus. Our findings demonstrate that baricitinib suppresses transcription of IFN-stimulated genes in non-infected cells, which is triggered by type I IFNs produced by infected cells, facilitating viral propagation. The pro-viral effect was influenced by viral load, inhibitor concentration, and structural characteristics of the compound. These results underscore the dual effects of JAK inhibitors: reducing inflammation while potentially exacerbating viral infections. Additionally, the findings highlight opportunities to leverage JAK inhibitors for viral research, vaccine production, and drug screening.

## Graphical Abstract

**Graphical Abstract:**
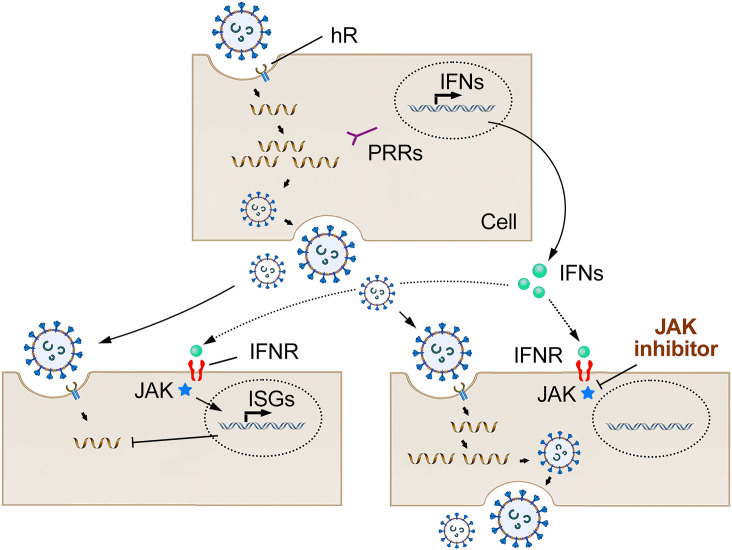

## Introduction

Janus kinase (JAK) inhibitors are pivotal in managing autoimmune and inflammatory conditions, offering targeted suppression of overactive immune pathways. By modulating the JAK–STAT signalling pathway, these drugs reduce chronic inflammation, improving outcomes in conditions like rheumatoid arthritis, psoriatic arthritis, and atopic dermatitis. Their therapeutic utility extends to haematological disorders and COVID-19 [1]. Currently, 10 JAK inhibitors have been approved by the FDA, while numerous investigational compounds are undergoing clinical trials or are in early stages of development [2–4].

JAK inhibitors vary in specificity, with some selectively targeting a single kinase (e.g. upadacitinib for JAK1 and deucravacitinib for TYK2) and others acting more broadly (e.g. baricitinib for JAK1/JAK2 and tofacitinib for JAK1/JAK3). These molecules typically bind to the ATP-binding site of JAKs, and their structural differences influence both their target selectivity and therapeutic applications [5].

Despite their effectiveness in managing inflammation, JAK inhibitors may impair antiviral defence, potentially increasing the risk of viral infections [6]. They are associated with elevated risks of upper respiratory tract infections, viral pneumonia, BK virus viraemia and viruria, viral gastroenteritis, and reactivation of viruses such as herpes simplex (HSV), herpes zoster (VZV), and hepatitis B (HBV) [4, 7–10]. For example, baricitinib has been linked to an increased incidence of VZV [11]. Patients receiving JAK inhibitors had a significantly higher likelihood of hospitalization or adverse outcomes, including mortality, compared to those treated with interleukin-6 (IL-6) inhibitors or tumour necrosis factor (TNF) blockers [12].

Animal studies provided compelling evidence of the impact of JAK inhibition on viral infections. For example, JAK inhibition during the early phase of Severe Acute Respiratory Syndrome Coronavirus 2 (SARS-CoV-2) infection has been shown to exacerbate kidney injury in mice by suppressing antiviral responses [13]. Similarly, tofacitinib treatment worsened outcomes in HSV infection models [14]. *In vitro* studies have also shown that JAK inhibitors could suppress antiviral responses and enhance replication of Vesicular Stomatitis Virus (VSV), Respiratory Syncytial Virus (RSV), Influenza A Virus (IAV), Measles Virus (MeV), Mumps Virus (MuV), Zika Virus (ZIKV), Human Herpesvirus 6A (HHV-6A), Reovirus (ReoV) [15–21]. Collectively, these findings suggest that JAK inhibitors may increase susceptibility to infections, though the detailed mechanism behind this adverse effect is not fully understood.

It is important to investigate the effects of JAK inhibitors on other viral infections, such as Rift Valley fever virus (RVFV). RVFV is a zoonotic pathogen that can infect humans, causing a wide range of clinical outcomes, from mild febrile illness to severe complications including hepatitis, haemorrhagic fever, and meningoencephalitis [22]. The primary organs affected by RVFV include the liver, lungs, eyes, and brain. Among these, neurological involvement, particularly encephalitis and other forms of brain damage, is considered a major contributor to mortality [23]. Understanding how JAK inhibitors influence the course of RVFV infection, especially in the context of neuroinflammation and viral replication in the central nervous system, may provide novel insights into pathogenesis and virulence of the rift valley fever.

Here, we bridge this gap by systematically elucidating the pro-RVFV effect of JAK inhibitors, identifying key factors such as drug concentration, chemical structure, and viral load that influence their impact on antiviral immunity. We showed how immunosuppressive properties of JAK inhibitors can compromise antiviral defenses, presenting risks during active RVFV and other viral infections.

## Materials and methods

### Small molecules, viruses, cells, and organoids

Abrocitinib (HY-107429), AT9283 (HY-50514), baricitinib (HY-15315), delgocitinib (HY-109053), fedratinib (HY-10409), filgotinib (HY-18300), itacitinib (HY-16997), oclacitinib (HY-13577), pacricitinib (HY-16379), ruxolitinib (HY-50856), tofacitinib (HY-40354), and upadacitinib (HY-19569) were obtained from MedChemExpress. To obtain 10 mM stock solutions, compounds were dissolved in dimethyl sulfoxide (DMSO; Sigma–Aldrich, Germany) or water. The solutions were stored at −20°C until use.

The recombinant rRVFVΔNSs::Katushka expressing the far-red fluorescent protein Katushka instead of the NSs protein (rRVFV) was generated as described previously [24]. The recombinant influenza A/PR/8-NS116-GFP strain (rIAV), expressing GFP instead of the effector domain of NS1, was generated as described previously [25]. Recombinant SARS-CoV-2/Wuhan-mCherry (rSARS-CoV-2), expressing mCherry, was generated as described previously [26]. Adenovirus (AdV) was derived from a nasopharyngeal swab sample collected at St. Olavs Hospital.

Human adenocarcinomic alveolar basal epithelial cells (A549, CCL-185), hTERT-immortalized retinal pigment epithelial cells (RPE, CRL-4000), human lung adenocarcinoma epithelial cells (Calu-3, HTB-55), Madin–Darby canine kidney cells (MDCK, CCL-34), and monkey Vero-E6 cells (Vero C1008, CRL-1586) cell lines were obtained from the ATCC. ACE2-expressing A549 cells (A549-ACE2) were from Chang *et al.* [27]. A549 and Vero-E6 cells were grown in Dulbecco’s Modified Eagle’s medium (DMEM; Gibco, Paisley, Scotland) supplemented with 100 U/ml penicillin and 100 μg/ml streptomycin mixture (pen/strep; Lonza, Cologne, Germany), 4.5 g/l (25 mmol/l) glucose, 1 mM l-glutamine, and 10% heat-inactivated fetal bovine serum (FBS; Lonza, Cologne, Germany). RPE and Calu-3 cells were grown in DMEM-F12 supplemented with 10% FBS, pen/strep. All cells were cultured at 37ºC with 5% CO_2_, 95% humidity, and passaged using 0.05% (v/v) Trypsin/EDTA (Gibco). Cells were tested mycoplasma negative throughout the work (MycoAlert Mycoplasma Detection Kit, Lonza).

Recombinant RVFV (rRVFV) and SARS-CoV-2 (rSARS-CoV-2) were amplified in a monolayer of Vero-E6 cells in growth media containing 1% FBS. Recombinant IAV (rIAV) was amplified in a monolayer of MDCK cells in virus growth media containing 0.2% bovine serum albumin (BSA, Sigma) and 0.2 μg/ml L-1-tosylamido-2-phenylethyl chloromethyl ketone (TPSK) trypsin (Sigma). Virus stocks were stored at −80°C.

Induced pluripotent stem cells (iPSCs) were used to generate retinal organoids as described previously [28, 29]. Brain organoids have been produced from material obtained from cancer patient with grade IV glioblastoma following the protocol by Jacob *et al.* [30].

### Drug treatment and virus infection of cells and organoids

Approximately 4 × 10^4^ cells were seeded in each well of 96-well/plate. After 24 h small molecules were added. Fifteen minutes later cells were infected. The infection of A549 and Vero-E6 cells with rRVFV was done in DMEM-based medium containing 1% FBS. The infection of RPE and Calu-3 cells with rRVFV was done in DMEM-F12-based media containing 1% FBS. The infection of A549 cells with AdV was done in DMEM-based medium containing 1% FBS. The infection of A549 cells with rIAV was done in DMEM-based media containing 0.2% BSA and 0.2 μg/ml TPSK-trypsin. The infection of A549-ACE2 cells with rSARS-CoV-2 was done in DMEM-based medium containing 1% FBS.

Forty-day-old retinal organoids were transferred from petri dishes to an ultralow attachment 96-well plates. One organoid was placed in each well in 96-well plate, with a total of 100 μl of media per well. Baricitinib (5 μM) or vehicle were added. Virus at multiplicity of infection (moi) 0.1 or mock was added after 15 min.

### Cell and organoid imaging, viability, and death assays

The cell and organoids were imaged using Evos FL Auto imaging system (Invitrogen). Cell viability and cytotoxicity were assessed using the CellTiter-Glo (CTG) and CellTox Green (CTxG) assays (Promega, Cat. nos. G9241 and G8741, respectively). Measurements were performed on a FluoStar Omega plate reader (BMG Labtech), followed by quantification of luminescence and fluorescence signals.

### Calculation of drug sensitivity scores

Drugs were added to cells in three-fold serial dilutions. Cells were infected with viruses at multiplicity of infection (moi) 0.1 or mock. The cell viability was measured using CTG assay after 48 h of infection. A drug sensitivity score (DSS) was calculated as a normalized version of the standard area under dose–response curve (AUC), with the baseline noise subtracted, and the normalized maximal response at the highest concentration (often corresponding to off-target toxicity):


(2) \begin{eqnarray*}
{\rm DSS }= \frac{{{\rm AUC }- t\left( {{{x}_{{\rm max}}} - {{x}_{{\rm min}}}} \right)}}{{\left( {100 - t} \right)\left( {{{x}_{{\rm max}}} - {{x}_{{\rm min}}}} \right){{{\log }}_{10}}A{\rm min}}},
\end{eqnarray*}

where activity threshold *t* equals 10%, and DSS is in the 0–50 range [31–34]. The difference (ΔDSS) between DSS (virus) and DSS (mock) was also calculated.

### Protein profiling

The proteome profiler human XL cytokine array kit (ARY022B), proteome profiler human apoptosis array kit (ARY009), and proteome profiler phospho-kinase array kit (ARY003C) from R&D Technologies were used to profile extracellular cytokines, intracellular proteins involved in apoptosis, and the phosphorylation status of proteins associated with various signalling pathways. All arrays were performed according to the manufacturer’s protocols.

### RNA sequencing

Total RNA was isolated from samples using NAxtra kit following the manufacturer’s protocol [35]. For library preparation, messenger RNA (mRNA) was enriched from the total RNA using oligo-dT beads to select for polyadenylated transcripts. The enriched mRNA was fragmented into smaller pieces, which were used for complementary DNA (cDNA) synthesis. First-strand cDNA synthesis was performed using random primers, followed by second-strand synthesis. The resulting cDNA fragments were end-repaired and adenylated at the 3′ ends, after which adaptors were ligated. Libraries were then amplified via polymerase chain reaction (PCR) and assessed for quality. The amplified libraries were circularized and further amplified to produce DNA nanoballs (DNBs). Sequencing was performed on the DNBSEQ Technology platform with a paired-end read length of 150 bp (PE150), using the DNBSEQ Eukaryotic Strand-specific mRNA library, as specified for DNBSEQ Eukaryotic Strand-specific Transcriptome Resequencing. Raw sequencing data were processed to remove low-quality sequences and adapter contamination. Reads containing 25% or more adapter sequence (allowing up to two mismatches) were discarded. Reads <150 bp were removed, as were those with an *N* content of 0.1% or more. Reads with stretches of a single nucleotide exceeding 50 bp were excluded. Additionally, reads where bases with a quality score <20 constituted 40% or more of the sequence were filtered out. The resulting high-quality reads were retained for downstream analysis, with quality scores reported using the Phred33 system.

RNA-seq reads were aligned using STAR (Spliced Transcripts Alignment to a Reference) version 2.7.11b to the reference human GRCh38 genome. Gene-level read counts were obtained using STAR’s built-in-quantMode GeneCounts function. Gene symbols and descriptions were assigned using the biomaRt package (v. 2.58).

Pathway enrichment analysis was conducted using the clusterProfiler R package (v. 4.8.3) to identify overrepresented biological pathways among differentially expressed genes [36]. Results were visualized as bubble plots, where the *x*-axis represented enriched pathways, the *y*-axis showed experimental comparisons, bubble size indicated the gene count (number of genes in each pathway), and bubble colour intensity reflected statistical significance (−log10(adjusted *P*-value)).

The RNA-seq data have been deposited in the NCBI Gene Expression Omnibus (GEO) under accession number GSE292720.

### qPCRs

Quantitative PCR (qPCR) was performed on the CFX Connect Real-Time PCR Detection System (Bio-Rad) using qScript^™^ 1-Step Virus ToughMix (Quantabio) and virus-specific primers and TaqMan probe (Supplementary Table S1). Quantitative RT-PCR (RT-qPCR) was performed using host-specific primers (Supplementary Table S2).

### Institutional review board statement

All experiments with viruses, cells, and organoids were performed in BSL2 and BSL3 laboratories in compliance with the guidelines of the national authorities (REK 392418). Standard operational procedures were submitted to the institutional safety committee (Nr. 57317).

## Results

### Potential pro-viral mechanism of action of the JAK inhibitors

**Figure 1.**
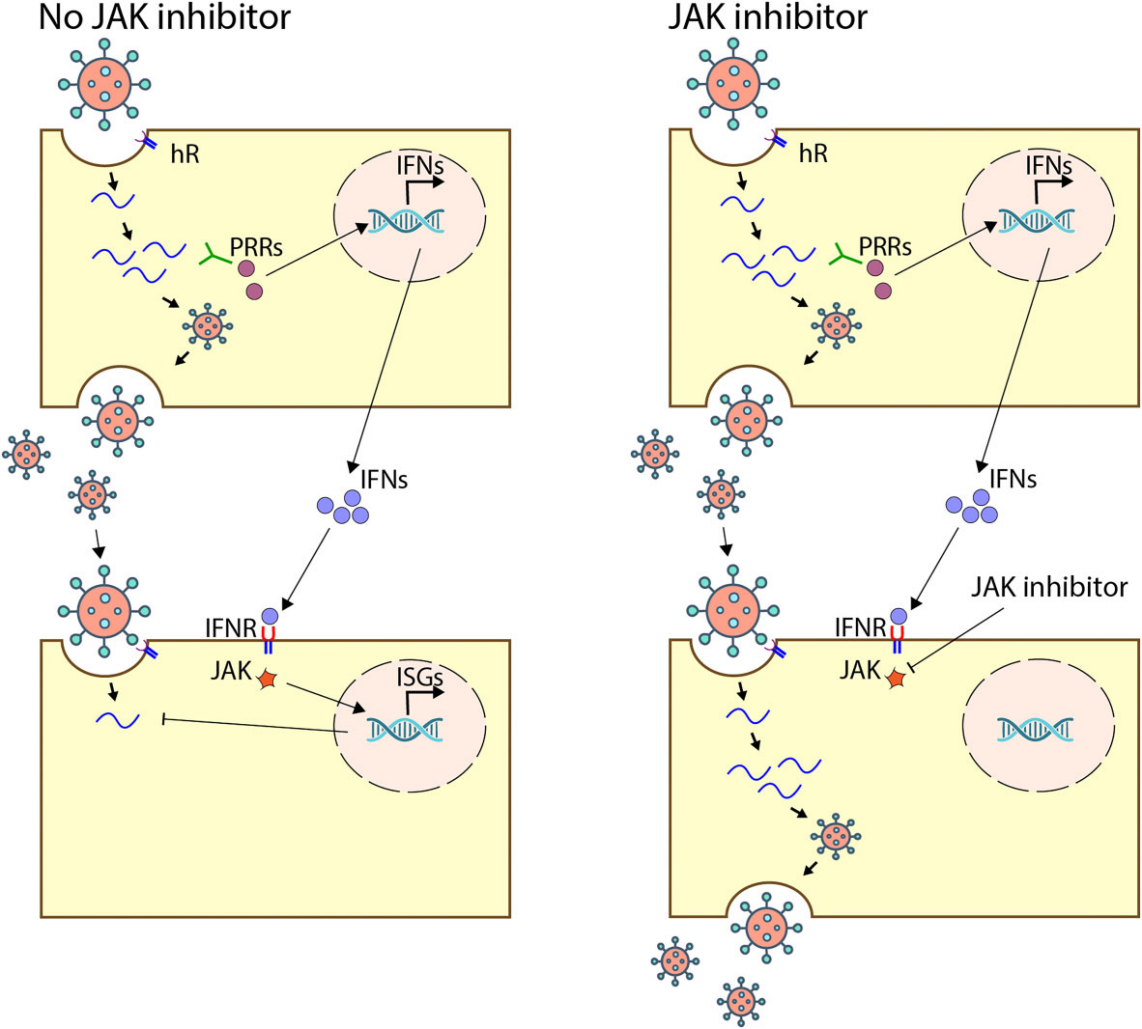
Potential pro-viral mechanism of action of the JAK inhibitors. Schematics showing how viral infections induce type I IFN synthesis in infected cell and its secretion. Secreted IFN triggers JAK–STAT pathway that activates ISG production, creating an antiviral state in uninfected cells (left panel). JAK inhibitors disrupt JAK–STAT pathway, removing innate immune barriers and enabling the amplification of viral particles in previously uninfected cells (right panel).

We hypothesized that certain JAK inhibitors, including approved ones (Supplementary Fig. S1), may suppress the interferon (IFN) response and facilitate the progression of viral infections through a straightforward mechanism. In this mechanism, viral infections stimulate the synthesis of IFNs in host cells, which are subsequently released into the extracellular space. IFNs then bind to receptors on the surface of uninfected cells and activate the JAK–STAT signalling pathway, leading to the synthesis of IFN-stimulated genes (ISGs) that protect the cells from potential infection. However, JAK inhibitors disrupt this protective response in uninfected cells, allowing viruses to infect new cells unimpeded and spread throughout the tissue (Fig. 1).

### Baricitinib enhances rRVFV infection in A549 cells

**Figure 2.**
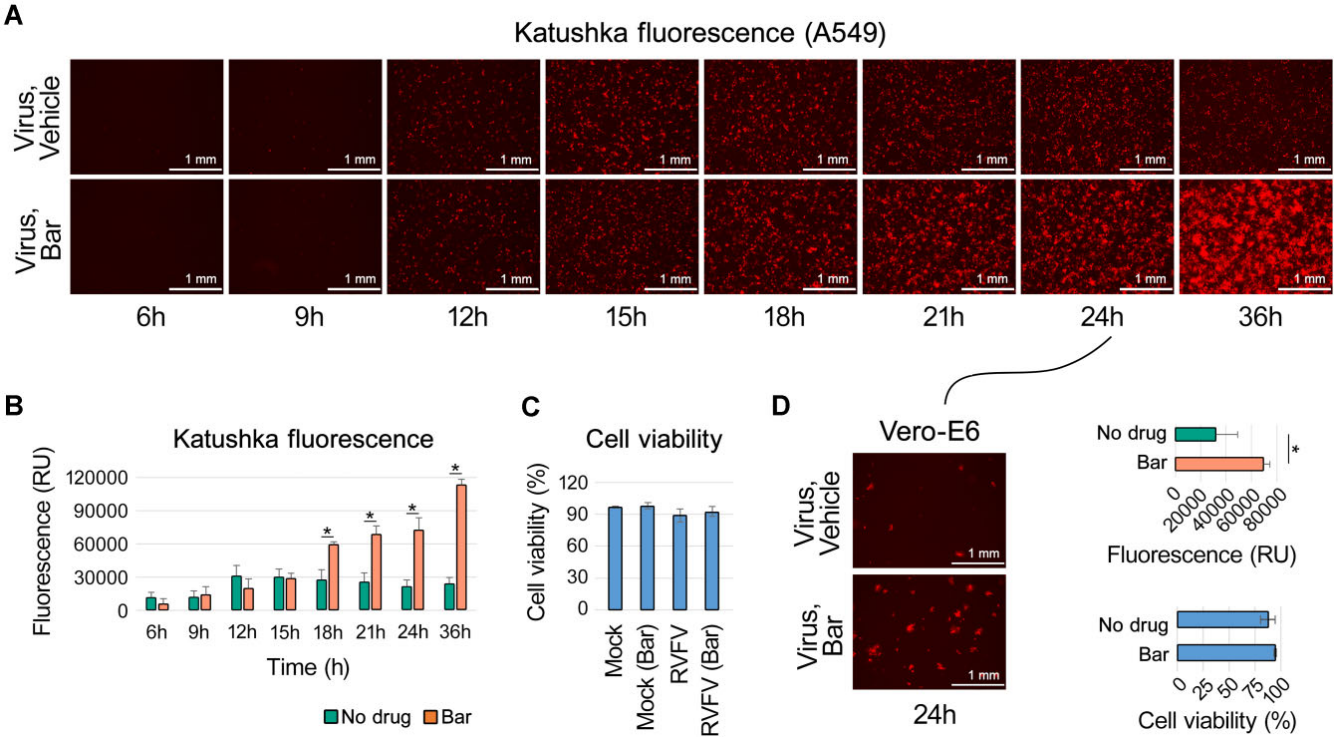
Baricitinib enhances the progression of rRVFV infection in A549 cells. (**A** and **B**) A549 cells were treated with either a vehicle or 5 μM baricitinib and infected with the rRVFV virus (moi 0.1). Images of the infected cells were captured, and fluorescence of far-red signal intensity was quantified. The fluorescence signal was normalized and expressed as mean ± SD (*n* = 3). (**C**) After 36 h, the cell viability of virus- and mock-infected, vehicle- and baricitinib-treated A549 cells was assessed using a CTG assay (mean ± SD, *n* = 3). (**D**) Supernatants from vehicle- and baricitinib-treated A549 cells were collected after 24 h of virus infection, diluted 1:10 000, and applied to Vero-E6 cells. Katushka reporter protein expression and cell viability were evaluated as described in panels (A–C). Statistical significance is indicated as *P < 0.05, determined using the Wilcoxon rank-sum test with alternative hypothesis “greater than.”

To prove the hypothesis, we first assessed the impact of baricitinib on rRVFV virus infection. A549 cells were treated with 5 μM baricitinib or a vehicle control (0.01% DMSO) and infected with the virus at a moi of 0.1. Fluorescent microscopy showed a time-dependent increase in the expression of the fluorescent Katushka protein in baricitinib-treated cells compared to the vehicle control (Fig. 2A and B). Notably, the treated cells remained viable after 36 hours post infection (hpi) (Fig. 2C).

We next evaluated the production of infectious virus particles. Supernatants from vehicle- and baricitinib-treated A549 cells were collected 24 h post-infection, diluted 1:1000, and applied to IFN-I-deficient Vero-E6 cells [37, 38], which are not sensitive to baricitinib during virus propagation (Supplementary Fig. S2A). Analysis of reporter protein expression and cell viability demonstrated that baricitinib treatment resulted in the production of higher levels of infectious virus particles (Fig. 2D).

We performed a time-of-addition experiment in A549 cells using the virus (Supplementary Fig. S3). The results showed that the baricitinib can be added between 0 and 6 h (corresponds to time of IFN induction) after infection to promote viral replication. These findings indicate that baricitinib enhances virus replication supporting our initial hypothesis.

### The pro-viral effect of JAK inhibitors is influenced by initial rRVFV load, concentration, and chemical structure of the small molecules

**Figure 3.**
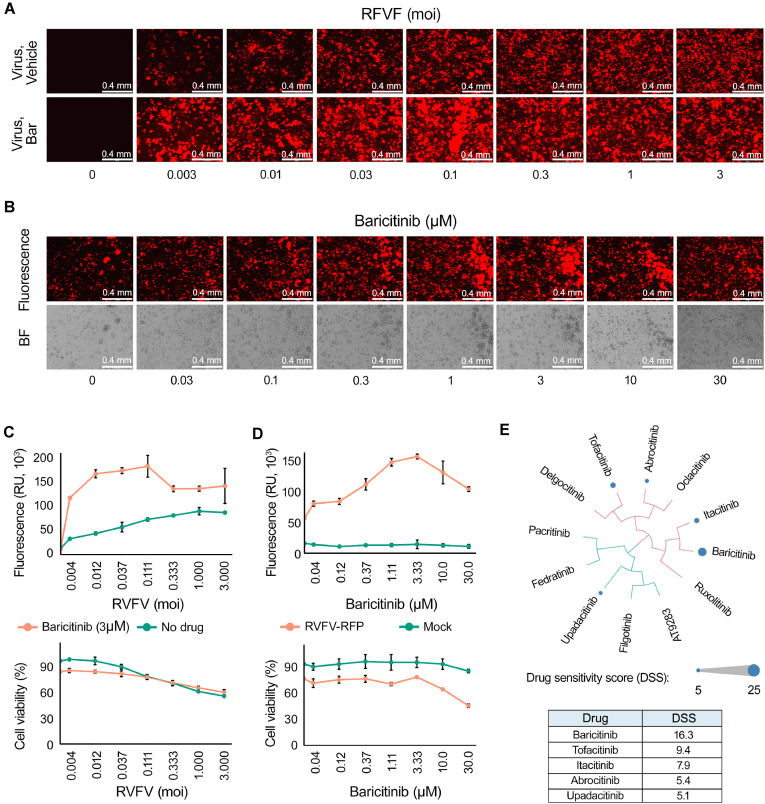
The pro-viral effect of JAK inhibitors is influenced by initial virus load, concentration and chemical structure of small molecules. (**A** and **C**) A549 cells were treated with either a vehicle or 5 μM baricitinib and infected with the rRVFV at varying moi. After 24 h, fluorescent and bright-field images were captured, and Katushka reporter protein expression and cell viability were quantified (mean ± SD, *n* = 3). (**B** and **D**) A549 cells were treated with vehicle or different concentrations of baricitinib and infected at an moi of 0.1. After 24 h, fluorescence and bright-field images were captured, and Katushka reporter protein expression and cell viability were analysed (mean ± SD, *n* = 3). (**E**) A549 cells were treated with varying concentrations of different JAK inhibitors. DSSs were calculated and visualized as bubbles in a SAR diagram. Bubble sizes represent DSS values, and compound similarities were determined using ECFP4 fingerprints and visualized through the D3 JavaScript library. The table (bottom panel) lists the exact DSS values for the five most active JAK inhibitors, where DSS is a normalized area under the dose–response curve (AUC, see “Materials and methods”).

To investigate the impact of viral load on the pro-viral effects of baricitinib, A549 cells were treated with either vehicle or baricitinib and subsequently infected with rRVFV at varying moi. After 24 h, fluorescent and bright-field microscopy images were captured, and Katushka reporter protein expression along with cell viability was measured. The results demonstrated that baricitinib exhibited a substantial pro-viral effect at moi <0.1 (Fig. 3A and C). These findings support the hypothesis that at least one round of viral replication is necessary to induce type I IFN production in infected cells, which allows baricitinib to block the IFN-mediated antiviral state in uninfected cells, thereby promoting subsequent rounds of viral replication.

To evaluate the concentration-dependent effects, A549 cells were treated with vehicle or increasing concentrations of baricitinib and infected with the virus at an moi of 0.1. After 24 h, fluorescence and bright-field images were captured, and both Katushka reporter protein expression and cell viability were analysed. Baricitinib displayed a dose-dependent enhancement of viral replication up to 10 μM concentration, when the drug became toxic (Fig. 3B and D).

The effects of 11 additional JAK inhibitors were subsequently assessed in A549 cells under conditions similar to those used for baricitinib. DSSs were calculated for each compound and visualized as bubbles in a structure–activity relationship (SAR) diagram (Fig. 3E), where bubble sizes correspond to DSS values. Among the tested inhibitors, upadacitinib, tofacitinib, itacitinib, and abrocitinib, in addition to baricitinib, demonstrated the ability to promote viral replication, whereas seven other JAK inhibitors did not exhibit this pro-viral effect at the tested concentrations (Supplementary Fig. S4).

Structural analysis revealed that the five active compounds shared a heterocyclic aromatic core, which serves as the molecular framework for specific interactions with the ATP-binding site of the JAK kinase domain (Supplementary Fig. S5). These findings suggest that the pro-viral effects of JAK inhibitors are influenced by both their chemical structure and concentration, with the heterocyclic aromatic core playing a pivotal role.

We screened 300 compounds from our in-house broad-spectrum antiviral library in combination with baricitinib [39, 40]. Our pilot screen revealed that A-1155463 and ABT-737 (both pro-apoptotic), as well as ozanimod (a sphingosine-1-phosphate receptor modulator), pimodivir (a PB2 inhibitor targeting influenza virus replication), and tiplaxtinin (a selective inhibitor of plasminogen activator inhibitor-1) enhanced the expression of the Katushka protein in rRVFV-infected A549 cells (Supplementary Fig. S6) [41–44]. By contrast, anisomycin (a protein synthesis inhibitor) suppressed rRVFV-mediated Katushka expression. These findings suggest that other compounds might further amplify the pro-viral effects of Jak inhibitors such as baricitinib.

### Baricitinib attenuates transcription of IFN-stimulated genes and modulates apoptotic, and signalling pathways in A549 cells during rRVFV infection

**Figure 4.**
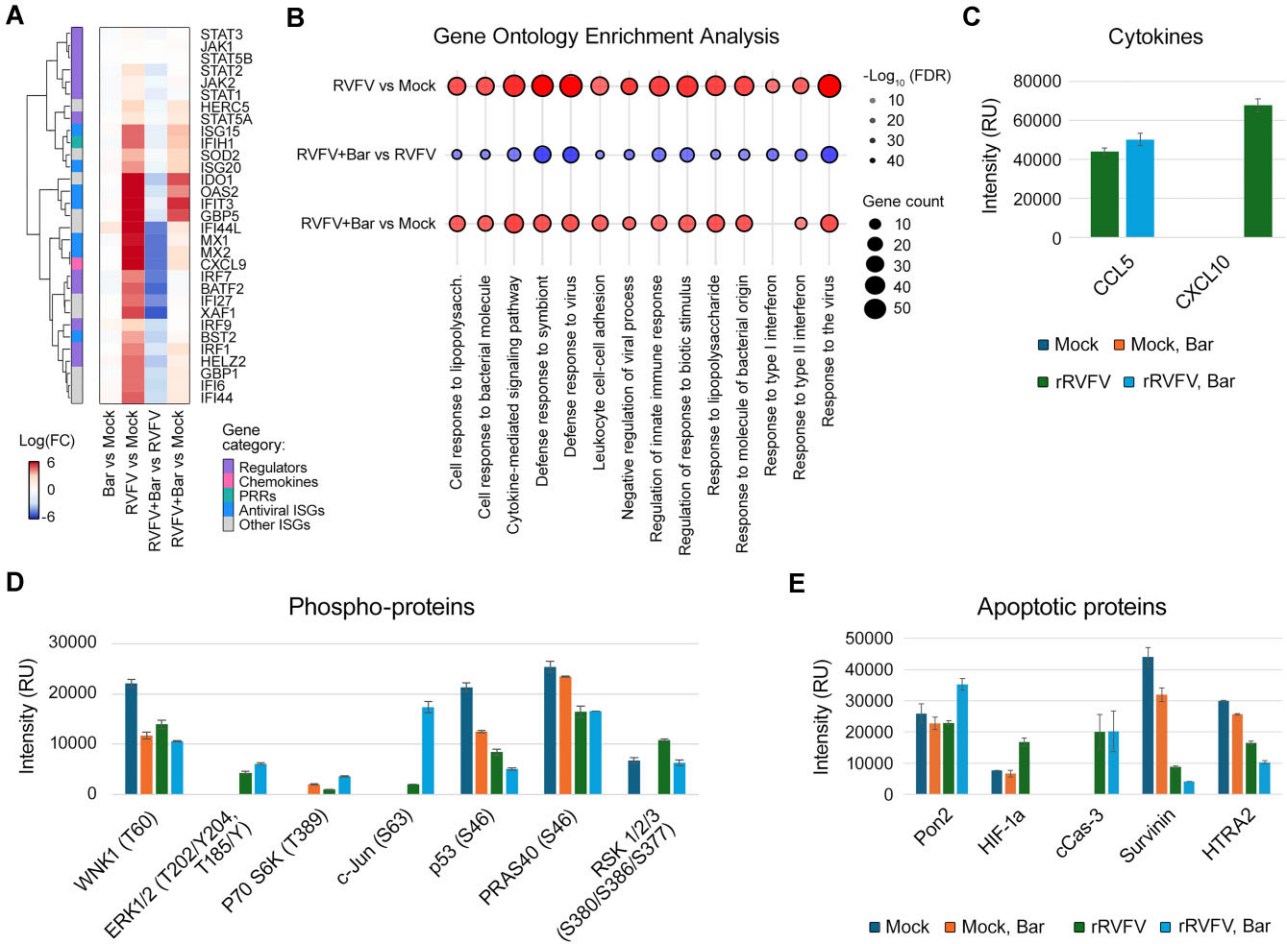
Baricitinib suppresses transcription of ISGs and imbalances signalling and apoptosis in rRVFV-infected A549 cells. (**A**) Cells were treated with 5 μM baricitinib or vehicle, and infected with the rRVFV or mock. After 8 h, RNA was extracted from cells and sequenced (mean ± SD, *n* = 3). Differential gene expression analysis was performed. Heatmap showing log_2_ fold changes (logFC) in expression of key IFN response genes across four experimental comparisons. Genes are clustered hierarchically based on expression patterns and annotated by functional categories. Columns represent distinct comparisons: “RVFV versus Mock” shows virus-induced changes; “Bar versus Mock” shows drug effects in uninfected cells; “RVFV + Bar versus RVFV” shows how the drug modulates infection response; and “RVFV + Bar versus Mock” represents the combined effect of virus and drug compared to control. (**B**) Bubble plot representing pathway enrichment analysis for three comparisons (rRVFV versus Mock, rRVFV/Bar versus rRVFV, and rRVFV/Bar versus Mock) is shown. Bubble size indicates the number of genes associated with each pathway (gene count), and bubble colour represents the statistical significance of enrichment, expressed as −log10(adjusted *P*-value). Red bubbles indicate higher pathway activity (upregulation), while blue bubbles suggest lower activity (downregulation or distinct regulation patterns). (**C**) Cells were treated as in panel (A). After 24 h, cell culture supernatants were collected, and cytokines were analysed using the human XL cytokine array; the altered protein levels were plotted (*n* = 2). (**D** and **E**) Cells were treated as in panel (A). After 24 h, cells were lysed; apoptotic and signalling proteins were analysed using the apoptosis and the phospho-kinase arrays, respectively; the altered protein levels were plotted (*n* = 2).

To investigate the effect of baricitinib on the host immune response to RVFV infection, A549 cells were treated with 5 μM baricitinib or vehicle, and infected with rRVFV or mock. RNA sequencing analysis revealed significant transcriptional changes during RVFV infection and baricitinib treatment. Differential expression analysis presented as a heatmap in Fig. 4A shows that rRVFV infection strongly upregulated key ISGs such as MX1, MX2, IFIT3, and IFI44L, compared to mock infection. Baricitinib treatment showed minimal effects on gene expression in uninfected cells but significantly attenuated the ISG response in RVFV-infected cells. The detailed statistical analysis of these differentially expressed genes is presented in volcano plots in Supplementary Fig. S7, which further illustrates the magnitude and significance of these transcriptional changes. Pathway enrichment analysis confirmed that rRVFV activated antiviral and immune signalling, while baricitinib suppressed activation of type I IFN pathway (Fig. 4B).

The cytokine array showed rRVFV-induced production of pro-inflammatory cytokines CCL5 and CXCL10, with baricitinib enhancing CCL5 while completely suppressing CXCL10 (Fig. 4C and Supplementary Fig. S8A). The phospho-kinase array showed that rRVFV activated MAPK signalling via ERK1/2 and c-Jun phosphorylation while suppressing p53 (S46), reducing apoptosis. Baricitinib amplified ERK1/2 and c-Jun activation but decreased survival-associated kinases such as WNK1 and RSK1/2/3 (Fig. 4D and Supplementary Fig. S8B). The apoptosis array revealed that rRVFV modulated cell death by increasing HIF-1a and cleaved Caspase-3 while reducing anti-apoptotic Survivin and HTRA2. Baricitinib partially reversed pro-apoptotic changes, suppressing HIF-1a and cleaved Caspase-3, but further reduced Survivin and HTRA2, indicating selective modulation of apoptosis during infection (Fig. 4E and Supplementary Fig. S8C).

These findings highlight baricitinib’s selective modulation of immune, apoptotic, and signalling pathways, amplifying MAPK activity while dampening IFN responses and survival pathways during RVFV infection.

### Baricitinib has pro-rRVFV effect in other cell types and organoids

RVFV infects multiple organs, including the lung, eyes, and brain, reflecting its ability to target diverse cell types [22, 45]. To investigate the pro-viral effects of baricitinib, experiments were conducted using other lung epithelial cells, retinal and brain cells as well as eyes and brain organoids.

In lung Calu-3, retinal RPE and brain GBP-1 cells, baricitinib or vehicle was added 15 min prior to infection with rRVFV or mock. Baricitinib enhanced viral replication in a dose-dependent manner, with significant effects observed, without detectable toxicity up to 10 μM (Supplementary Fig. S2B–D).

**Figure 5.**
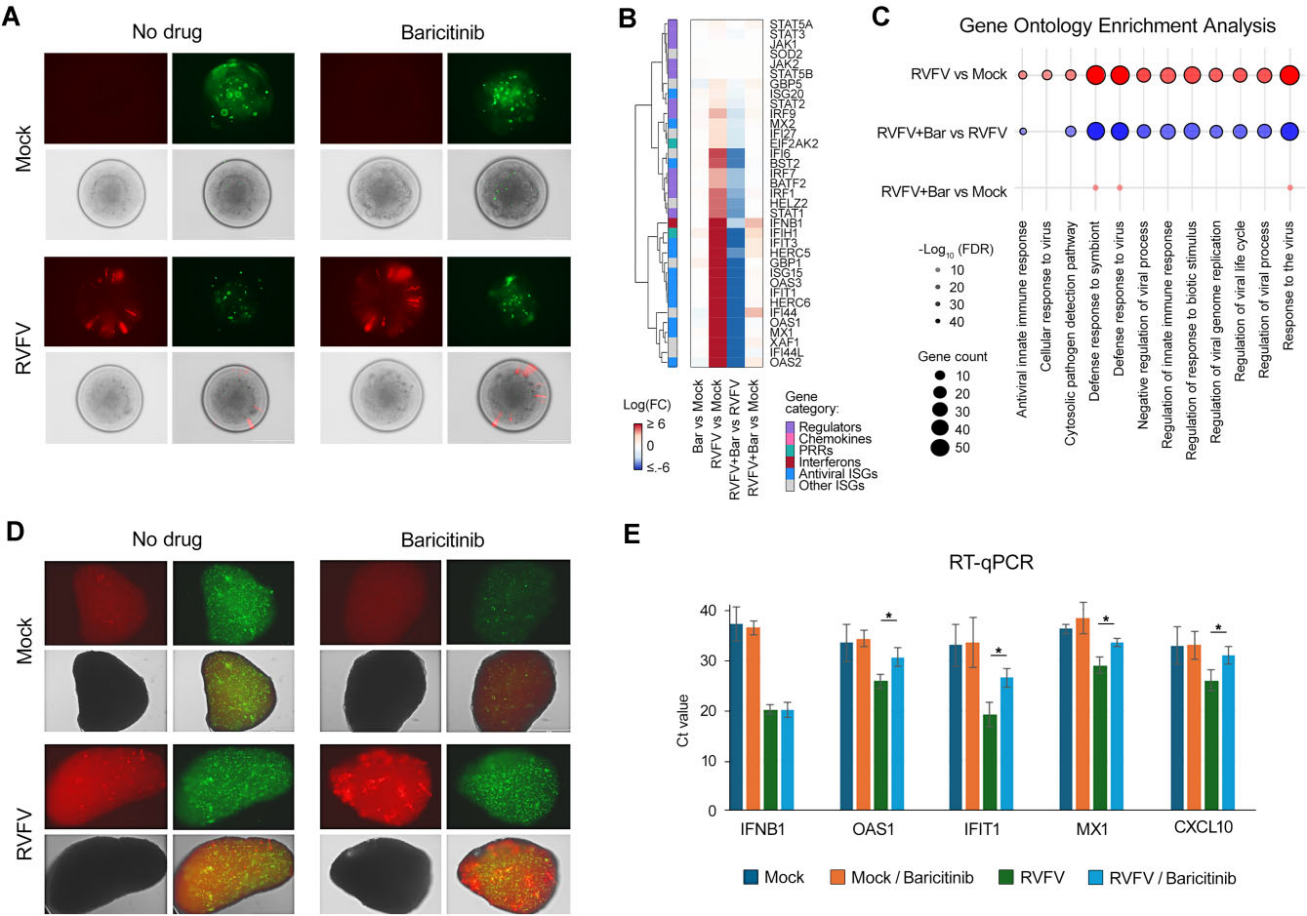
Effect of baricitinib on retinal (hROs) and brain (hGBOs) organoids. (**A**) hROs were treated with 5 μM baricitinib or vehicle, and infected with the rRVFV or mock. After 48 h, microscopic images of organoids were taken. Red channel—far-red fluorescent protein Katushka, green channel—CellToxGreen; scale bar: 400 mm. (**B**) Organoids were treated as in panel (A) (mean ± SD, *n* = 3). After 24 h, RNA was extracted and sequenced. Differential gene expression analysis was performed. Heatmap showing log_2_ fold changes (logFC) in expression of key IFN response genes across four experimental comparisons. Genes are clustered hierarchically based on expression patterns and annotated by functional categories. Columns represent distinct comparisons: “RVFV versus Mock” shows virus-induced changes; “Bar versus Mock” shows drug effects in uninfected cells; “RVFV + Bar versus RVFV” shows how the drug modulates infection response; and “RVFV + Bar versus Mock” represents the combined effect of virus and drug compared to control. inrRVFV/Vehicle and rRVFV/Baricitinib versus Mock is shown. The *x*-axis denotes significantly enriched pathways, while the *y*-axis represents the conditions compared. Bubble size corresponds to the number of genes involved in each pathway (gene count), and bubble colour indicates the direction of regulation (red for upregulated pathways and blue for downregulated pathways). (**D**) hGBOs were treated with 5 μM baricitinib or vehicle and infected with the rRVFV or mock. After 48 h, microscopic images of organoids were taken. Red channel—mCherry, green channel—CellToxGreen; scale bar: 400 mm. (**E**) Organoids were treated as in panel (D) (mean ± SD, *n* = 3). After 48 h, RNA was extracted, and RT-qPCR analyses were performed. Statistical significance between virus-infected and virus-infected drug-treated hGBOs is indicated as *P < 0.05, determined by the Wilcoxon rank-sum test with alternative hypothesis “greater than.”

Given RVFV’s ability to infect RPE and GBP-1 cells, experiments were extended to retinal and glioblastoma organoids. Human retinal (hROs) and glioblastomal (hGBOs) organoids were treated with 5 μM baricitinib or vehicle, followed by rRVFV or mock infection. After 48 h, fluorescence imaging showed that baricitinib substantially increased RVFV infection, as demonstrated by heightened Katushka reporter protein expression (Fig. 5A and D, and Supplementary Fig. S9).

Differential gene expression analysis in hROs at 24 h post-infection revealed a robust upregulation of antiviral genes (e.g. *IFIT1*, *IFIT2*, *MX1*, and *OAS2*) and pathways related to antiviral innate immunity and viral replication in rRVFV versus mock comparisons. Baricitinib treatment suppressed these responses, downregulating key IFN-stimulated genes such as *IFIT1*, *MX1*, *STAT1*, and *OAS1* in rRVFV/baricitinib versus rRVFV comparisons, indicating inhibition of IFN signalling (Fig. 5B and C, and Supplementary Fig. S10).

RT-qPCR analysis of RNA isolated from hGBOs at 48 h post-infection revealed a robust upregulation of *IFNB1* and *IFNB1*-stimulated *IFIT1*, *MX1*, *CXCL10*, and *OAS1*. Baricitinib treatment suppressed the expression of ISGs but not IFNB1 (Fig. 5E and Supplementary Fig. S10).

These results demonstrate that baricitinib enhances rRVFV infection while suppressing immune responses in both lung-, eye- and brain-derived models. This highlights baricitinib’s pro-viral effects and its potential to modulate type I IFN-mediated antiviral pathways across different cell types and organ systems.

### Pro-viral effect of baricitinib

**Figure 6.**
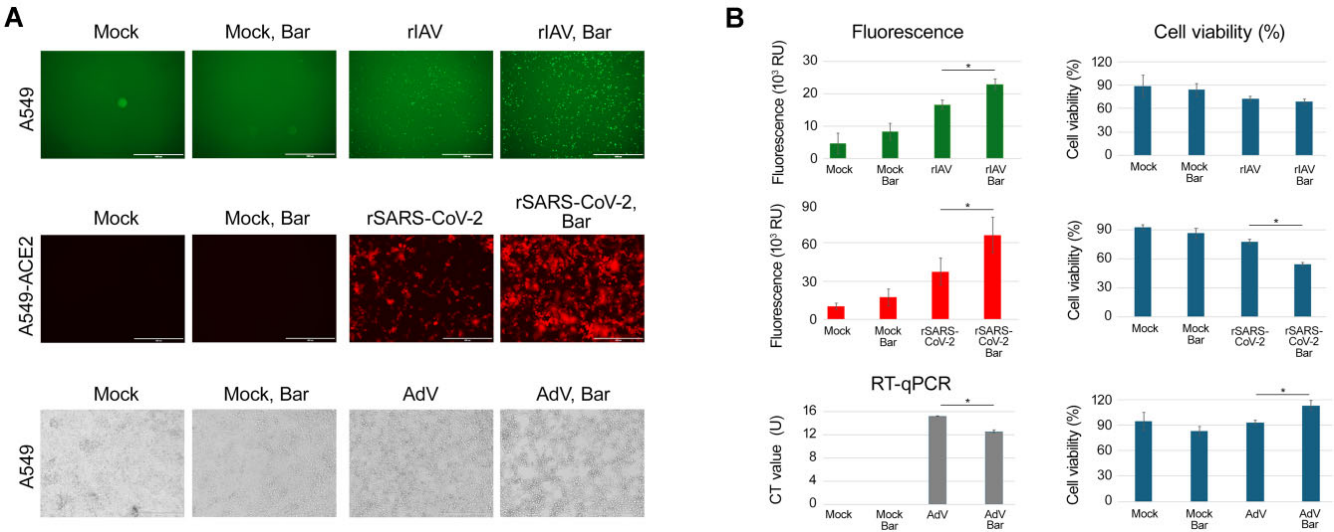
Pro-influenza (rIAV), coronavirus (rSARS-CoV-2), and adenovirus (AdV) effects of baricitinib in A549 or A549-ACE2 cells. (**A** and **B**) Cells were treated with 5 μM baricitinib or vehicle and infected with viruses at an moi of 0.1. After 48 h of rIAV infection, 24 h of rSARS-CoV-2 infection and 72 h of AdV infection images of cells were captured. The red (mCherry) and green (eGFP) fluorescence signals were quantified, normalized, and expressed as mean ± SD (*n* = 3). Cell viability was assessed using a CTG assay (mean ± SD, *n* = 3). qPCR analysis of AdV in the media was performed. The *C*_T_ values were expressed as mean ± SD, *n* = 3. Cell viability was assessed using a CTG assay (mean ± SD, *n* = 3). Statistical significance is indicated as *P < 0.05, determined by the Wilcoxon rank-sum test with alternative hypothesis “greater than.”

To further investigate the pro-viral effects of baricitinib, A549 or A549-ACE2 cells were treated with 5 μM baricitinib or vehicle and infected with rIAV, rSARS-CoV-2, or AdV at an moi of 0.1. In A549 cells infected with rIAV, fluorescence microscopy revealed a marked increase in GFP expression in baricitinib-treated cells compared to vehicle controls, indicating enhanced influenza virus replication (Fig. 6A). Similarly, in A549-ACE2 cells infected with rSARS-CoV-2, baricitinib treatment resulted in increased mCherry fluorescence, suggesting amplified replication of rSARS-CoV-2 (Fig. 6B). In A549 cells infected with AdV, qPCR analysis showed increased secretion of adenoviruses in the media of baricitinib-treated cells (Fig. 6B). This provides further evidence of baricitinib’s ability to promote viral replication by disrupting type I IFN signalling.

## Discussion

Understanding both the therapeutic benefits and potential side effects of medications is crucial. Certain drugs, commonly prescribed for the treatment of other diseases, can inadvertently influence viral infections, potentially altering patient outcomes. For example, anticancer Bcl-xL inhibitors could facilitate killing of animals with virus infections [42, 43]. Atorvastatin, candesartan, and hydroxocobalamin could target influenza A virus–host cell interaction and affect the transcription and metabolism of infected cells [46].

In this study, we demonstrated that infection with IFN-sensitive rRVFV induced the synthesis of type I IFNs in A549 cells. These IFNs were released extracellularly, bound to IFNR on uninfected cells, and activated the JAK signalling, leading to the production of ISGs. The ISGs provided antiviral protection to the remaining uninfected cells. However, baricitinib disrupted this protective mechanism by inhibiting JAK signalling, by preventing ISG production. This inhibition allowed the virus to spread more effectively.

The pro-viral effect of baricitinib was influenced by several factors, including initial viral load, drug concentration, structural characteristics of the inhibitor, cell type, and the addition of other small molecules. Moreover, baricitinib treatment modulated apoptotic and kinase signalling pathways during rRVFV infection in A549 cells. This JAK inhibitor also demonstrated pro-viral effects in retinal and glioblastoma cells and retinal and glioblastoma organoids. Beyond rRVFV, baricitinib enhanced the replication of other viruses, including rIAV, rSARS-CoV-2, and AdV, highlighting a broad pro-viral effect across multiple viruses and cellular models.

Our study has several limitations, as it primarily relies on *in vitro* models, high drug concentration and includes IFN-sensitive viruses such as rRVFV, rIAV, and rSARS-CoV-2. These models may not fully capture the complexity of immune responses *in vivo*. Nevertheless, previous in vitro studies with other viruses, including VSV, RSV, IAV, MeV, MuV, ZIKV, HHV-6A, and ReoV, have also demonstrated that JAK inhibitors can suppress antiviral responses and enhance viral replication [15–21]. Furthermore, *in vivo* studies have confirmed that JAK inhibition exacerbates infections caused by SARS-CoV-2 and HSV-1. Additionally, clinical evidence suggests that patients with autoimmune conditions who are on JAK inhibitors face an increased risk of hospitalization and death from COVID-19 [12–14], further supporting our findings.

The ability of JAK inhibitors to enhance viral replication presents valuable opportunities in research and biotechnology. Specifically, incorporating JAK inhibitors into cell culture systems can significantly improve diagnostic sensitivity by amplifying low-titer viral samples and boosting the yields of attenuated viruses for vaccine production (EP3801584 and WO2024/003718). Additionally, JAK inhibitors hold promise in antiviral drug discovery as combination agents, enabling viral propagation to identify compounds targeting different stages of viral replication. Furthermore, these inhibitors can enhance the production efficiency of complex oncolytic viruses, achieving higher titers for therapeutic applications. JAK inhibitors can enhance viral vector-based gene delivery by reducing immune responses and improving transduction efficiency. Thus, JAK inhibitors have the potential to revolutionize vaccine production, streamline drug discovery, and improve diagnostic sensitivity for challenging low-titer viral samples.

Understanding the dual nature of JAK inhibitors is critical for optimizing their clinical use. While these drugs provide significant therapeutic benefits in reducing inflammation, they may inadvertently enhance viral propagation by suppressing type I IFN signalling and ISG production. Future research should explore the potential for combination therapies that pair JAK inhibitors with antiviral agents to balance immunosuppression and infection risk. Moreover, the structural characteristics identified in this study can guide the design of next-generation JAK inhibitors with enhanced pro-viral activity.

Many commonly prescribed drugs could modulate virus-host cell interactions, thus contributing to the morbidity and mortality of patients with virus infections. Further studies are needed to understand the side effects of the drugs during infectious and perhaps other diseases.

In conclusion, JAK inhibitors, such as baricitinib, can suppress type I IFN-mediated antiviral responses, leading to increased viral propagation. This highlights the need for careful consideration when administering these drugs to patients with active viral infections, as they may inadvertently facilitate viral proliferation. Conversely, this property of JAK inhibitors can be harnessed in vaccine development to enhance viral yields in controlled settings, antiviral drug screening in IFN-sensitive cells and organoids as well as in oncolytic virus research to enhance virus-mediated death of tumours.

## Supplementary Material

ugaf017_Supplemental_File

## Data Availability

All data supporting the findings of this study are available within the manuscript and its supplementary materials. Any additional data will be made available by the corresponding author upon reasonable request. The RNA-seq data have been deposited in the NCBI Gene Expression Omnibus (GEO) under accession number: GSE292720.
